# Tyrannical leadership predicts psychotropic use over time: a longitudinal study of healthcare workers in Chile

**DOI:** 10.3389/fpsyg.2026.1804035

**Published:** 2026-04-09

**Authors:** Elisa Ansoleaga, Ståle Valvatne Einarsen, Bernardita Reyes-Martínez

**Affiliations:** 1 Faculty of Psychology, Universidad Diego Portales, Santiago, Chile; 2 Department of Psychosocial Science, University of Bergen, Bergen, Norway; 3 Department of Psychology, Universidad de Concepción, Concepción, Chile

**Keywords:** healthcare workers, occupational stress, psychotropic medication, tyrannical leadership, workplace violence

## Abstract

**Introduction:**

Healthcare workers are exposed to intense organizational pressures and demanding interpersonal dynamics that may undermine psychological wellbeing. Tyrannical leadership—characterized by hostile, controlling, and humiliating supervisory behaviors-represents a particularly harmful organizational stressor in healthcare settings. Under such conditions, psychotropic medication may be used as a coping response to sustained psychological strain. This study examined whether exposure to tyrannical leadership predicts subsequent psychotropic drug use among healthcare workers.

**Methods:**

A longitudinal panel study was conducted among healthcare workers employed in high-complexity public hospitals in Chile. Two waves of data were collected 10 months apart. The analytic sample included 683 participants who completed both measurements. Tyrannical leadership exposure was assessed using a validated scale. Psychotropic medication use was measured through self-reported intake of hypnotics, anxiolytics, or antidepressants. Logistic regression models were used to estimate crude and adjusted associations between baseline tyrannical leadership and psychotropic drug use at follow-up, controlling for baseline medication use, sex, and key psychosocial work stressors, including effort-reward imbalance and emotional demands.

**Results:**

At follow-up, 39.8% of participants reported psychotropic medication use. In crude models, exposure to tyrannical leadership at baseline was associated with approximately twice the odds of psychotropic use at follow-up. In the fully adjusted model, baseline psychotropic use was the strongest predictor of subsequent consumption, with nearly tenfold higher odds of continued or increased use. After adjustment, tyrannical leadership remained significantly associated with psychotropic drug use, doubling the odds of consumption. Effort-reward imbalance also showed a significant association, whereas sex and emotional demands were not significant predictors.

**Discussion:**

These findings identify tyrannical leadership as a significant and potentially preventable organizational risk factor linked to increased psychotropic medication use among healthcare workers. By highlighting leadership as a modifiable determinant of mental health outcomes, the study underscores the importance of organizational interventions aimed at improving supervisory practices in high-stress healthcare environments.

## 1 Introduction

Healthcare workers are exposed to multiple work-related stressors (Rink et al., 2023; Hamama et al., 2022) that can negatively affect their job performance, psychological wellbeing, and overall quality of care they provide (Babapour et al., 2022; Deng et al., 2019).

Among the most significant stressors are interpersonal stressors within work teams, particularly relationships with supervisors and any exposure to tyrannical or abusive leadership (Rink et al., 2023). Exposure to such deficient leadership is consistently found to associated with lower job satisfaction and wellbeing in workers in both western countries as well as in Africa and Asia (Shih et al., 2023; Musinguzi et al., 2018). Similar patterns have been observed in Latin America, where tyrannical leadership has been identified as an important psychosocial risk factor for workers' mental health, particularly in high-stress environments such as healthcare institutions (Palma and Ansoleaga, 2022).

Tyrannical leadership involves sustained exposure to behaviors that undermine employees' wellbeing, motivation, or job satisfaction, while not necessarily undermining the organization's goals, values and mission (Einarsen et al., 2011). Although such may appear aligned with organizational goals and strategies, they often achieve these outcomes at the expense of their subordinates' wellbeing. Tyrannical leaders manipulate, humiliate, abuse and intimidate subordinates. Employing the parallel term, abusive supervision, Tepper (2000) defines it as “subordinates' perceptions of the extent to which supervisors engage in the sustained display of hostile verbal and non-verbal behaviors, excluding physical contact”. Indirectly, this may again affect the organization negatively and even the care and treatment of patients (Bamforth et al., 2023). Yet, the most consistent negative outcome is found among subordinates who may become highly stressed, with lower self-esteem and self-efficacy and experiencing psychosomatic and mental health problems.

Tyrannical leadership can also be understood within the broader literature on workplace aggression and psychological violence. Research on workplace bullying has for instance shown that persistent exposure to hostile leadership behaviors may constitute a form of organizational aggression that undermines employees' psychological safety and wellbeing (Einarsen et al., 2011, 2020). Such behaviors often involve humiliation, intimidation, or systematic degradation of subordinates, placing tyrannical leadership conceptually close to other forms of workplace mistreatment examined in occupational health research. From this perspective, destructive leadership, in our case tyrannical behaviors, can be interpreted not only as ineffective management practices but also as a potential form of workplace psychological violence with important implications for employees' mental health.

Because many employees cannot afford to stop working despite experiencing psychological distress and a bad working situation, some may rely on psychotropic medications to cope with work-related stressors (Milner et al., 2019). Drugs can be used to manage symptoms or as a coping mechanism to avoid dealing with their accompanying emotions (Gerich and Lehner, 2023). Exposure to stressful environments has been identified as a risk factor for the consumption of psychotropics (Dinis-Oliveira and Magalhães, 2020), and particularly among health care workers as a high-risk group (Maggioli, 2020). The use and abuse of psychotropics have shown a correlation with workplace accidents, absenteeism, and presenteeism, as well as developing dependency on the substance (Dinis-Oliveira and Magalhães, 2020), with both licit and illicit psychotropics. The associated psychotropic consumption can also become iatrogenic to the workers, further affecting their mental, physical, and social life (Cerecedo Pérez et al., 2013; Ansoleaga, 2015).

From a stress and coping perspective (Lazarus and Folkman, 1984), exposure to tyrannical leadership may constitute a chronic psychosocial stressor that can undermine employees' psychological well-being, creating fair, uncertainty and prolonged anxiety (see also Fosse et al., 2019). According to the Transactional Model of Stress and Coping, individuals facing persistent stressors may adopt various coping strategies to manage the resulting emotional and physiological strain. In this context, the use of psychotropic medication may reflect attempts to alleviate stress-related symptoms such as anxiety, sleep disturbances, or depressive symptoms. Within occupational health research, such adverse leadership behaviors can be understood as psychosocial job stressors that contribute to strain responses, consistent with frameworks such as the Job Demands–Resources model (Bakker and Demerouti, 2007). From this perspective, psychotropic medication use may represent a form of symptom management associated with work-related stress exposure, rather than solely an indicator of pre-existing mental health disorders. Thus, tyrannical leadership may function as a workplace stressor that contributes to psychological strain, which in turn may increase the likelihood of psychotropic medication use as a strategy to manage stress-related symptoms. At the same time, it can also be interpreted as a proxy indicator of underlying mental health problems, as these medications are commonly prescribed for conditions such as anxiety, depressive symptoms, and sleep disturbances.

Beyond the general stress–coping framework, several psychological mechanisms may help explain how exposure to tyrannical leadership may translate into psychotropic medication use. Repeated exposure to hostile or humiliating supervisory behaviors may generate chronic psychological strain, emotional exhaustion, and sleep disturbances, which are well-established precursors of anxiety and depressive symptoms (Einarsen et al., 2011; Nielsen and Einarsen, 2012). Over time, such strain responses may lead workers to seek pharmacological strategies to manage distress or maintain functioning at work. In this sense, psychotropic medication use may reflect attempts to regulate stress-related symptoms or maladaptive coping responses associated with prolonged exposure to destructive leadership behaviors (Gerich and Lehner, 2023).

Recent studies have shown how exposure to a violent workplace may be a risk factor for the use of psychotropics (Conway et al., 2025), yet employing cross-sectional data. In addition, the health sector has particular structural and cultural characteristics that can increase the risk of mental health problems and the consequent use of psychotropic drugs. Healthcare organizations are traditionally characterized by hierarchical, rigid structures with clear power differences between occupational groups and hierarchical levels (Hamui-Sutton et al., 2014). In this culture, employees may become particularly vulnerable to how leaders exercise their authority (Curry et al., 2015). Therefore, tyrannical leaders may pose a danger to the healthcare system as a whole, affecting the mental health of their subordinates and cascading across the organization and its patients (Burgos Moreno and Paravic Klijn, 2003).

However, other variables related to mental health (Conway et al., 2025), for example, a stressful life event, or other psychosocial factors at work, such as emotional demands, may act as work stressors with possible implications for mental health. Therefore, it is necessary to examine the long-term impact of tyrannical leadership on workers' mental health while controlling for such confounding factors. This study therefore aims to examine whether exposure to tyrannical leadership at time 1 (T1) predicts psychotropic medication use at time 2 (T2), adjusting for baseline psychotropic use and potential confounders. So, we hypothesized that healthcare workers exposed to tyrannical leadership at time 1 would have higher odds of psychotropic use at time 2, after controlling for confounders. This study contributes to the existing literature by employing a longitudinal design and controlling for key psychosocial confounders relevant to healthcare workers. While much of the research on psychological violence at work, including workplace bullying and tyrannical leadership/abusive supervision, has been conducted in Western countries, the present study was conducted in a sample of healthcare workers in high complexity hospitals in Chile. In this study, psychotropic medication refers to the use of hypnotics, anxiolytics, and antidepressants, which are commonly prescribed to treat sleep disturbances, anxiety, and depressive symptoms. These medications differ in their mechanisms of action and clinical indications but are frequently used to manage stress-related psychological symptoms. The measure used in this study captures self-reported use of these medications, regardless of whether or not they were medically prescribed (Skurtveit et al., 2008).

## 2 Materials and methods

The research approach of this study was quantitative, non-experimental, and a longitudinal panel, employing a questionnaire. Two measurements were collected at 10-month aparts in a sample of workers from high-complexity public health hospitals in Chile's three most populous regions. Participants represented diverse occupational roles within the healthcare system, including technical staff (38.7%), administrative personnel (14.2%), other health professionals (13.3%), auxiliary staff (13.8%), nursing staff (10.1%), and physicians (10.0%), reflecting the hierarchical and multidisciplinary structure typical of healthcare organizations.

**Figure 1:**
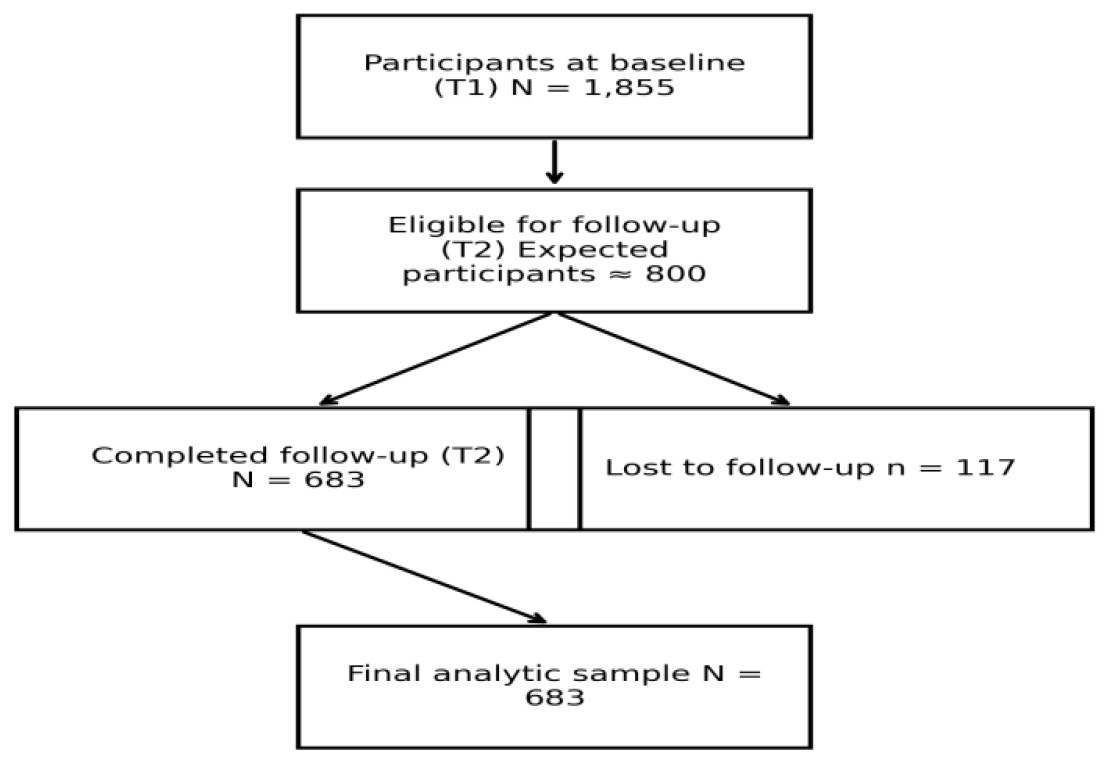
Flow diagram illustrating participant selection and follow-up between baseline (T1) and follow-up (T2).

The sample was probabilistically selected and stratified by gender and job position, with 1,855 participants at time 1 (68% response rate). The second measurement reached 683 of the 800 participants estimated at time 2, resulting in an 85.5% response rate in the longitudinal sample. The participant selection process and follow-up are illustrated in Figure 1. A total of 117 participants were in the follow-up, representing an attrition rate of only 14.6%. Baseline characteristics were compared between participants included in the longitudinal analyses and those lost to follow-up. No significant differences were observed for sex, occupational category, education, effort–reward imbalance, or exposure to tyrannical leadership. Participants retained in the study were slightly older and more likely to report baseline psychotropic use and higher emotional demands. Baseline psychotropic use and emotional demands were included as covariates in the regression models. The data collection was conducted by a data collection company through face-to-face surveys and mobile devices to systematize responses.

Exposure to tyrannical leadership was measured using four items from the Spanish version of the Destructive and Constructive Leadership scale (Aasland et al., 2010; González-Santa Cruz and Ansoleaga, 2023). The ítems are: 1. Has he/she humiliated you or other workers if they do not meet the expected standards? 2. Has he/she made gestures (e.g., grimaces, looks, gestures) toward you or other workers to express dissatisfaction with your efforts or those of others? 3. Have they spread incorrect information about you or your coworkers with the intention of damaging your position or theirs in the institution? 4. Have they scolded you over the phone, hung up, or sent a rude email because they thought you had done a poor job?. The scale on tyrannical Leadership has a satisfactory reliability (Cronbach's alpha = 0.72 at T1 and 0.75 at T2). Results were reduced to a dichotomous variable of exposure to tyrannical leadership at T1 (dichotomized: 1 = exposed, 0 = unexposed), as reported by Aasland et al. (2010). Using this classification criterion, participants who reported that their leaders presented one or more tyrannical behaviors with a frequency of “usually”, “almost always”, or “always” over the last 6 months were considered as exposed to tyrannical leadership.

Psychotropic consumption was assessed using three single items measuring self-reported use of hypnotics, anxiolytics, and antidepressants. These items were combined into a dichotomous variable indicating the use of any psychotropic (1 = use, 0 = no use). Stimulant medications (e.g., methylphenidate-type prescriptions) were not included, as their prescription and distribution are highly regulated in the Chilean healthcare system. Therefore, the measure focuses on the psychotropic medications most used to manage symptoms such as anxiety, sleep disturbances, and depressive symptoms.

Additionally, several psychosocial factors were evaluated as potential confounders, including sex, emotional demands, and effort–reward imbalance, all measured at T1.

Emotional demands were measured with a subscale of “SUSESO/ISTAS21”, the Chilean version of the Copenhagen Psychosocial Questionnaire (COPSOQ) (Candia et al., 2020), which includes quantitative psychological demands, emotional demands associated with them, and the hiding of emotions. The instrument utilizes a five-point Likert scale from 0 (never) to 4 (always), reporting α = 0.78.

Effort-reward imbalance was measured with the Spanish version of Siegrist et al. (2009) effort-reward imbalance scale, validated by Ansoleaga et al. (2014). The ten-item scale measures the perceived imbalance between effort and reward at work. It uses a five-point Likert scale from 0 (strongly disagree) to 4 (strongly agree), with α = 0.72.

The questionnaire also included items on sex, age, occupation, etc.

### 2.1 Statistical analysis

Changes in prevalence between T1 and T2 were assessed using McNemar's test for paired categorical data. Logistic regression models were used to estimate the association between exposure to tyrannical leadership at T1 and psychotropic medication use at T2.

Potential confounders were evaluated sequentially. Each variable was introduced into the crude model separately, and its confounding role was assessed using three criteria. First, the variable had to show a statistically significant association with the outcome based on a Wald test in the adjusted model. Second, model fit improvement was evaluated using changes in Akaike's Information Criterion (AIC) and Bayesian Information Criterion (BIC), considering meaningful improvements in model fit when AIC decreased by more than 2 units and BIC by more than 6 units. Third, the variable had to produce a change of at least 10% in the tyrannical leadership coefficient (log-odds) after adjustment.

A variable was retained as a confounder only if it met these criteria. Based on this procedure, emotional demands, effort–reward imbalance, and sex were included in the adjusted models. Finally, a fully adjusted model incorporating all identified confounders was estimated.

In the analyses, the outcome corresponds to psychotropic medication use at T2 while statistically adjusting for baseline use at T1. Therefore, the models estimate the association between tyrannical leadership and psychotropic use at follow-up rather than incident use or dosage escalation.

### 2.2 Software

All analyses were conducted in Stata 15.0 (Stata Corp, 2017).

## 3 Results

### 3.1 Sample and descriptive statistics

The analysis included *N* = 683 healthcare workers with complete data on psychotropic use at both Times 1 and 2. A majority of the sample are female, 60.76% (*n* = 415), with 39.24% (*n* = 268) males.

The mean age was 38.1 years (SD = 10.9; range 18–67) and participants represented a broad range of occupational roles within the healthcare system, including technical staff (38.7%), administrative personnel (14.2%), auxiliary staff (13.8%), other health professionals (13.3%), nursing staff (10.1%), and physicians (10.0%). This distribution reflects the multidisciplinary and hierarchical structure typical of healthcare organizations. In terms of educational attainment, most participants reported having completed technical education (45.2%) or university education (32.4%), followed by secondary education (12.6%) and postgraduate studies (9.7%), while only a very small proportion reported basic education (0.2%).

Table 1 describes the prevalence of the exposure, outcome, and confounders included. As shown, the prevalences are mostly stable over time. Of these participants, as many as 39.82% (*n* = 272) reported psychotropic medication in Time 2.

**Table 1:** Prevalence of tyrannical leadership, psychotropic drug use and organizational confounders at T1 and T2.

Prevalences	T1 % (*N*)	T2 % (*N*)	*p*-value
Tyrannical leadership	20.79 (142)	21.38 (146)	0.75
Organizational dimensions			
• Emotional demands (↑ median)	52.56 (359)	51.68 (353)	<0.001
• Effort-reward imbalance	57.83 (395)	58.13 (397)	0.90
Psychotropic use (yes)	34.99 (239)	39.82 (272)	0.009

*N* = 683; *P*-values correspond to McNemar tests for paired categorical data comparing. T1 and T2 observations.

The prevalence of tyrannical leadership remained stable between T1 and T2 (McNemar χ^2^(1) = 0.10, *p* = 0.75). A significant change in emotional demands between T1 and T2 was observed (McNemar χ^2^(1) = 16.54, *p* < 0.001). Effort-reward imbalance remained stable between T1 and T2 (McNemar χ^2^(1) = 0.02, *p* = 0.90). Psychotropic drug use increased significantly between T1 and T2 (McNemar χ^2^(1) = 6.85, *p* = 0.009).

Table 2 shows the results of a logistic regression that examined the likelihood of psychotropic use at Time 2 as a function of exposure to tyrannical leadership at Time 1 and baseline psychotropic use estimated in Stata 15.0.

**Table 2:** Crude association between tyrannical leadership at T1 and psychotropic use at T2.

Psychotropic use T2	Odds ratio (OR)	95% CI	*p*-value
Tyrannical leadership (yes vs. no)	2.41	[1.56–3.73]	<0.001
Baseline psychotropic use (yes vs. no)	9.88	[6.82–14.32]	<0.001
Constant	0.23	[0.17–0.29]	<0.001

_cons estimate baseline odds. *N* = 683.

The model included *N* = 683 observations and demonstrated good fit: Wald χ^2^(2) = 202.61, *p* < 0.001, and a McFadden's pseudo-*R*^2^ of 0.2206.

Employees exposed to tyrannical leadership had higher odds of psychotropic use at T2 compared with those not exposed (OR = 2.41; 95% CI [1.56–3.73]; *p* < 0.001). In addition, participants who reported psychotropic use at baseline showed substantially higher odds of psychotropic use at follow-up (OR = 9.88; 95% CI [6.82–14.32]; *p* < 0.001).

Table 3 presents the results of a logistic regression model predicting the odds of psychotropic use at time 2, including potential confounders. The model included exposure to tyrannical leadership, baseline psychotropic use (psychotropic_T1), sex (female vs. male), emotional demands, and effort-reward imbalance.

**Table 3:** Adjusted Logistic Regression Predicting Psychotropic Use at Time 2 (*N* = 683).

Psychotropic use T2	Odds ratio (OR)	95% CI	*p*-value
Baseline Psychotropic Use T1 (yes vs. no)	9.58	[6.55−14.02]	<0.001
Tyrannical Leadership T1 (yes vs. no)	2.18	[1.38−3.45]	0.001
Emotional demands T1 (yes vs. no)	1.28	[0.87−1.87]	0.201
Effort-Reward Imbalance T1 (yes vs. no)	1.49	[1.02−2.16]	0.035
Female (vs. male)	1.17	[0.80−1.71]	0.398
*Constant*	0.14	[0.09−0.22]	<0.001

Statistics of the model: *N* = 683; LR χ^2^(5) = 202.61; *p* < 0.001; Log likelihood = −357.87239; Pseudo *R*^2^ = 0.2206.

After adjustment for sex, emotional demands, and effort–reward imbalance, exposure to tyrannical leadership remained significantly associated with psychotropic use at follow-up (OR = 2.18; 95% CI [1.38–3.45]; *p* = 0.001). Baseline psychotropic use showed the strongest association with the outcome (OR = 9.58; 95% CI [6.55–14.02]; *p* < 0.001). Effort–reward imbalance was also associated with higher odds of psychotropic use (OR = 1.49; 95% CI [1.02–2.16]; *p* = 0.035), whereas emotional demands and sex were not statistically significant predictors.

Overall, the full model explained 22.06% of the variance in psychotropic use (pseudo-R^2^ = 0.2206), indicating a substantive role for both exposure to tyrannical leadership and baseline medication status in predicting subsequent psychotropic consumption.

In conclusion, exposure to tyrannical leadership was associated with approximately twice the odds of psychotropic use at follow-up, independent of sex and other psychosocial work stressors.

## 4 Discussion

This investigation fills a critical gap in occupational health research by empirically linking exposure to destructive leadership in the form of tyrannical leadership, also denoted as abusive supervision, to psychotropic medication use among healthcare workers, controlling for other psychosocial stressors. Although numerous studies have documented how job stressors undermine employee wellbeing, few have quantified the pharmacological consequences of tyrannical supervisory behaviors in health care settings. By showing that exposure to tyrannical leadership more than doubles the odds of psychotropic use, we highlight the potential downstream implications of leader-subordinate interactions for healthcare professionals' coping responses.

After adjusting for baseline medication use and the measured confounders (sex, emotional demands, and effort–reward imbalance), tyrannical leadership was associated with psychotropic medication use at follow-up, independent of these factors. Notably, a substantial proportion of participants who reported psychotropic use at baseline also reported use at follow-up, highlighting the persistence of medication use over time and underscoring the potential value of leadership-focused interventions to improve psychosocial working conditions.

So, our findings underscore the dual importance of leadership behavior and individual and organizational factors in predicting psychotropic medication uptake among healthcare workers in high-complexity hospitals. Some remarks are that tyrannical leadership is a risk factor; yet the baseline psychotropic use is the strongest predictor of psychotropic use in the future; there are no gender differences, and the organizational dimension in practice lost significance after adjusting the model.

Consistent with international evidence (Shih et al., 2023; Musinguzi et al., 2018; Wang et al., 2022), exposure to a tyrannical supervisor—characterized by intimidation, interactional injustice, and verbally abusive behavior was associated with 2.18 times higher odds of psychotropic use. This aligns with stress-and-coping theory (Lazarus and Folkman, 1984), whereby authoritarian leadership represents a chronic organizational stressor that may precipitate coping strategies (Yagil et al., 2011) and maladaptive coping like pharmacological management of anxiety or sleep disturbance. One plausible interpretation is therefore that tyrannical leadership acts as a chronic psychosocial stressor that may contribute to psychological strain, emotional exhaustion, and sleep disturbances, well-established precursors of anxiety and depressive symptoms. Our findings should then be interpreted considering that psychotropic medication use may function as a proxy indicator of mental health problems, as medications such as anxiolytics, antidepressants, and hypnotics are typically prescribed to treat anxiety, depressive symptoms, or sleep disturbances, and such use may also function as a coping strategy. When systematic exposure to tyrannical/abusive leadership practices generates chronic psychological strain reactions in the form of anxiety and depressive symptoms, these strain responses may increase the likelihood that exposed employees may turn to pharmacological strategies to manage their distress. Such use may even serve to maintain one's work performance and overall functioning at work under such strain. Hence, the use of psychotropic medication can be used to regulate symptoms or as maladaptive coping responses following exposure to a tyrannical and abusive leader (Gerich and Lehner, 2023).

Our findings are quite in line with those of a recent Danish large-scale study combining data from surveys among the general working population and registry data on hospital-redeemed psychotropic drug prescriptions, showing that employees who reported exposure to workplace bullying had a 1.43 (CI: 1.35–1.53) higher risk of such drugs over some years. This study, however, did not control for other work-related stressors, only basic demographics, and did not distinguish between mistreatment from superiors and that from colleagues (Conway et al., 2025). Tyrannical leadership and its effects on subordinates may be particularly prominent in the healthcare sector due to the highly hierarchical relationships between workers and leaders (Essex et al., 2023). Interventions should therefore target leadership practices to mitigate downstream impacts on employee mental health, as described above.

In line with international evidence (Ettorre et al., 1994), prior psychotropic use emerged as the strongest predictor (OR = 9.58), highlighting the persistence of underlying mental-health conditions or established coping patterns. Clinicians and occupational health providers should monitor this subgroup closely, as they remain at elevated risk for continued or increased reliance on medication. Also, the use of psychotropic drugs can affect the quality of the service (Kerr et al., 2020; Cypher, 2024).

While effort–reward imbalance remains associated with higher odds of psychotropic use, contrary to expectations, self-reported emotional demands lost significance after adjustment, potentially due to shared variance with tyrannical leadership or baseline medication use. Likewise, sex differences were not observed, indicating that, in this context, organizational factors overshadow demographic influences. The lack of sex differences was again consistent with the Danish study on workplace bullying (Conway et al., 2025). Other authors have neither found gender differences in the association between abusive supervision and alcohol consumption (Zhou et al., 2018) nor in mental health problems between health workers (Palma and Ansoleaga, 2022), maybe because the health sector is an over-feminized work context.

### 4.1 Theoretical and practical implications

From the perspective of workplace violence prevention, our findings suggest that tyrannical leadership should be considered a form of psychosocial aggression and a relevant target for prevention efforts in healthcare organizations. Interventions that strengthen respectful leadership, improve reporting and response procedures for workplace mistreatment, and address organizational norms that tolerate hostile supervisory behaviors may contribute to reducing psychosocial harm and its downstream mental health consequences.

By elucidating the impact of tyrannical leadership on pharmacological coping, this study extends models of workplace stress to include leadership as a determinant of mental health trajectories. Organizations should incorporate leadership-development programs that cultivate supportive supervision alongside routine mental-health screening for employees with prior psychotropic use. Monitoring critical incidents of tyrannical leadership behaviors and developing strategies to minimize their effects would also be useful for the affected workers.

In a field strained by high-stakes decisions and systemic pressures, tyrannical leadership compounds risks, eroding workforce resilience, and may even be threatening patient treatment and care.

However, as any damaging effects of those leaders are preventable, this underscores the importance of studying and understanding this relationship. Organizations can and should address tyrannical leaders through improved selection procedures and training programs, improved policies and complaints procedures, yet also focusing on the potential preventive effect of cultural changes involving stronger norms and values for managers, as well as developing evidence-based strategies to protect healthcare workers' mental health.

### 4.2 Strengths, Limitations and Further Research

The present study has some notable strengths. It has a longitudinal/prospective design based on a relatively large sample. In addition, we controlled for other highly likely confounding stressors in the work environment and used well-established, validated scales. Yet, there are also some noteworthy limitations. First, the observational design limits causal inferences. Furthermore, unmeasured confounding variables (e.g., personality, prior trauma) may bias estimates. Although several psychosocial work stressors were considered as potential confounders and evaluated using predefined criteria, other potentially relevant covariates—such as age, occupational category, shift work, and indicators of psychological distress—were not included in the final models. These variables were not incorporated either because they did not meet the predefined criteria for confounding in the model selection procedure and/or were not consistently available. Nevertheless, residual confounding cannot be entirely ruled out and should be considered when interpreting the observed associations. Future studies should examine these factors more explicitly to further clarify the mechanisms linking adverse leadership behaviors and psychotropic medication use. Second, the study used self-reported measures. Psychotropic medication use was assessed through questionnaire items referring to the use of hypnotics, anxiolytics, and antidepressants. As with all self-reported measures, this approach may introduce reporting bias, as participants may underreport or misreport medication use. In addition, the survey did not distinguish between medically prescribed use and potential non-prescribed consumption. Therefore, the measure should be interpreted as an indicator of psychotropic medication use rather than a precise assessment of clinically prescribed treatment. Nevertheless, self-reported medication use is commonly employed in population-based studies and provides a useful proxy for mental health-related pharmacological treatment (Skurtveit et al., 2008). Future studies could benefit from linking survey data with prescription or administrative health records to further validate medication use, as done in register-based studies using national prescription data (see also., Conway et al., 2025). Regarding sample generalizability, our findings are based on healthcare workers in Chile; therefore, replication in other cultural and occupational settings must be carefully addressed. Furthermore, longitudinal designs with multiple waves could provide a clearer understanding of temporal ordering and potential mediation pathways (e.g., whether tyrannical leadership increases emotional exhaustion, which then elevates psychotropic use). Experimental studies evaluating leadership training interventions would further inform causal mechanisms and prevention strategies.

## 5 Conclusions

In a sample of 683 healthcare workers, exposure to tyrannical leadership was associated with more than double the odds of psychotropic use over a subsequent follow-up period, even after adjusting for baseline medication use, gender, emotional demands and effort–reward imbalance. Prior psychotropic users had nearly ten-fold greater odds of continued or escalated use. Other covariates, such as sex and emotional demands, did not reach significance. The complete model accounted for 22.06 % of the variance in psychotropic use.

Hence, our results highlight tyrannical leadership as a modifiable organizational predictor of psychotropic medication uptake, emphasizing the critical role of supervisory behavior in safeguarding employees' mental health. From a practical standpoint, these results provide actionable insights for hospital administrators and health system policymakers. Implementing leadership-development programs that emphasize supportive supervision and curtail tyrannical behaviors may not only strengthen workplace climate but also reduce reliance on pharmacotherapy—thereby lowering institutional costs and enhancing staff resilience. More broadly, by positioning leadership as a modifiable organizational determinant of psychotropic use, this study provides a conceptual and empirical framework for other high-stress healthcare environments in which supervisor conduct directly influences employee mental health.

## Data Availability

The datasets presented in this article are not readily available because the data will be available upon request and with the authors permission. Requests to access the datasets should be directed to Elisa Ansoleaga, maria.ansoleaga@udp.
